# Measuring ADRD Knowledge and Opinions of African American Families in South Carolina

**DOI:** 10.1093/geroni/igaf122.1178

**Published:** 2025-12-31

**Authors:** Lena Simon, Abigail Stephan, Angie Sardina, Jeff Meadows, Marsha Hampton, Lesley Ross, Alyssa Gamaldo

**Affiliations:** Clemson University, Clemson, South Carolina, United States; Clemson University, Clemson, South Carolina, United States; Clemson University, Clemson, South Carolina, United States; Clemson University Institute for Engaged Aging, Clemson, South Carolina, United States; Clemson University, Clemson, South Carolina, United States; Clemson University, Clemson, South Carolina, United States; Clemson University, Clemson, South Carolina, United States

## Abstract

Black Americans are at a disproportionate risk for being diagnosed with Alzheimer’s disease or related dementias (ADRD), which can negatively impact care planning. Although Black families are a pivotal source for care planning, limited research has explored ADRD knowledge and influential factors to seek ADRD care among Black families. This study (1) explores the relationships between perceptions of aging/ADRD research participation and ADRD care planning, and (2) explores changes in ADRD knowledge following participation in the facilitated focus group session among family members about their ADRD care planning. The sample included 40 South Carolinians (age range: 45–89, 97.5% black, 82.5% female), who completed surveys on perceptions of aging/ADRD research participation, and ADRD care planning prior to the focus group session. The Basic Knowledge of Alzheimer’s Disease (BKAD) scale was assessed before and 1-week after the focus group. Results prior to the focus group indicated that seeking care for a family member with suspected ADRD was positively correlated with recommending future research studies to others (r = .596, p = <.001). Recommending participating in aging research to others was also positively correlated with higher anticipation the focus group session would be helpful (r = .48, p = .002). Additionally, ADRD knowledge significantly increased between the pre- (M = 24.44, SD = 4.47) and 1-week post-focus group assessment (M = 26.27, SD = 3.7; p < .05). These preliminary analyses suggest that focus groups might be an acceptable method to increase ADRD literacy, conversation, and research participation for Black Americans.

